# Interleukin-1β induced vascular permeability is dependent on induction of endothelial Tissue Factor (TF) activity

**DOI:** 10.1186/1479-5876-3-37

**Published:** 2005-09-30

**Authors:** Markus Puhlmann, David M Weinreich, Jeffrey M Farma, Nancy M Carroll, Ewa M Turner, H Richard Alexander

**Affiliations:** 1 Surgery Branch, National Cancer Institute, National Institutes of Health, Bethesda, Maryland, 20892, USA

**Keywords:** videomicroscopy, inflammation, neovasculature, endothelial tissue

## Abstract

IL-1β is a pleotropic cytokine that may mediate increased procoagulant activity and permeability in endothelial tissue during inflammatory conditions. The procoagulant effects of IL-1β are mediated through induction of tissue factor (TF) but its alterations on vascular permeability are not well characterized. We found that IL-1β induced a rapid and dose-dependent increase in TF activity in human umbilical vein endothelial cells (ECs) under routine culture conditions. However, IL-1β caused a rapid and marked increase in permeability across confluent EC monolayers using a two-compartment *in vitro *model only in the presence of factor VIII-deficient plasma that was completely abrogated by neutralizing anti-TF antibody pre-treatment. *In vitro *permeability was associated with loss of EC surface expression of VE-cadherin and contraction of F-actin cytoskeletal elements that resulted in EC intercellular gap formation. These data demonstrate that IL-1β induces marked changes in permeability across activated endothelium via a TF dependent mechanism and suggest that modulation of TF activity may represent a strategy to treat various acute and chronic inflammatory conditions mediated by this cytokine.

## Introduction

IL-1β is a pleotropic 17.5 kD cytokine, secreted primarily by monocytes and macrophages, that mediates the pathophysiology of various acute and chronic inflammatory conditions. High levels of circulating IL-1 have been identified in experimental models of endotoxic shock and acute bacterial infection and increased gene expression of IL-1 has been identified in tissues at sites of experimentally induced inflammation [1,2]. Clinically, high levels of circulating IL-1 have been identified in patients with acute bacterial infections and elevated levels of IL-1 have been detected in the diseased articular tissues of patients with chronic rheumatoid arthritis [3,4]. In experimental models of endotoxin or *E. coli *induced shock, immune complex colitis, cancer progression, cachexia, and non-specific inflammation, IL-1 blockade significantly ameliorates the pathophysiological host response in these conditions [5-9]. Of note, administration of recombinant IL-1ra is used clinically to ameliorate the symptoms of severe rheumatoid arthritis [10].

Although IL-1 receptors are widely expressed on different cell types, the protein appears to exert many of its physiological actions through effects on endothelial tissue. IL-1 induces endothelial cell (EC) production of cytokines such as IL-8, IL-6 and TNF, multiple cytokine receptors, adhesion molecules, growth factors, matrix metalloproteinases, and coagulation factors such as tissue factor, fibrinogen, urokinase plasminogen activator, type 1,2 plasminogen activator and protease nexin 1 [11-14].

Two sentinel homeostatic functions of vasculature are initiation of coagulation via the extrinsic clotting cascade and regulation of vascular permeability. Increased vascular permeability most likely represents the initial event in pathological or reparative inflammation or angiogenesis by allowing efflux of plasma proteins into interstitium to serve as a provisional matrix for circulating inflammatory cells or activated endothelium [15]. IL-1 is known to induce a procoagulant phenotype on endothelial tissue primarily through induction of TF [16,17] and has been shown to alter endothelial cell monolayer permeability in *in vitro *models [18,19]. However, its effects on vascular permeability *in vivo *have not been consistent and the mechanisms for these effects are not known [20-22]. For example, in lapine models, systemic recombinant IL-1 has been shown to induce shock and significant pulmonary vascular injury manifested by marked perivascular pulmonary edema [20,23]. However, in rodent or guinea pig models others have not demonstrated any independent effects of aerosolized or systemically administered IL-1 on pulmonary vascular permeability or injury [21,22].

The present studies were performed to characterize the inflammatory properties of IL-1 as mediated by alterations in permeability across endothelial monolayers *in vitro*. The data indicate that IL-1 induces both procoagulant effects and permeability via a single TF dependent mechanism and suggest that TF may represent a novel target for acute or chronic inflammatory conditions mediated by IL-1.

## Materials and methods

### Cell culture

Human umbilical vein ECs (hUVECs) were obtained from Clonetics (Clonetics #CC 2519) and propagated at 37°C in a 5% CO_2 _incubator in EGM-2 media (Clonetics #CC-3156 and #CC-4176) according to the manufacturer's instructions but without addition of the VEGF-supplement. Cells in the experiments were passaged for a maximum of 5 generations. MC-38, a non-metastatic methylcholanthrene-induced murine adenocarcinoma was maintained in Dulbecco's modified Eagle's medium (DMEM) (Biofluids, Rockville, MD) supplemented with 10% heat-inactivated fetal calf serum, 2 mM glutamine and 50 U/mL penicillin/streptomycin.

### *In vitro *permeability assay

In-vitro permeability was quantitated by spectrophotometric measurement of Evans Blue-bound albumin across functional hUVEC monolayers using a modified 2-compartment chamber model as previously described in detail.  Briefly, hUVECs were plated (8 × 10^5^/ well) in 1 μm PET Transwell filter inserts (Falcon #35 3102) using EGM-2 (as described above). On day 3, cells were washed and treated with EBM-2 (Clonetics #CC-3156) with 2% BSA with or without IL-1β with various concentrations as indicated. Except during time course experiments, cytokine exposure was kept constant at 2 hours (at 37°C, 5% CO_2_). Cells were briefly washed with PBS/ Ca^2+ ^and Mg^2+^. Fresh EBM-2 including 1% Factor VIII-deficient plasma (George-King Biochemical) (where indicated) was added and incubated for an additional 1 hour at 37°C, 5% CO_2_. In experiments evaluating TF-blocking antibodies, designated wells were incubated first with a 1:100 dilution of antibody (American Diagnostica, #4509) and PBS/1% BSA. Inserts were washed with PBS/ Ca^2+ ^and Mg^2+ ^before adding 1.5 mL of Evans Blue (EB) bound to 0.1% BSA in PBS in the upper chamber. Two mL PBS/ Ca^2+ ^and Mg^2+ ^was added to the lower chamber in which absorbance of EB was determined after 1 hr using a spectrophotometer (620 nm). Experiments were performed in triplicates and repeated multiple times.

### Tissue factor assay

Tissue factor activity was determined using a 1-stage coagulation assay. HUVECs were plated (8 × 10^5^/ well) in a 6-well plate and incubated for 3 days. Cells were washed with PBS and treated with various concentrations of IL-1β (in EBM-2/ 2% BSA) for 2 hours at 37°C, 5% CO_2_. After this incubation, HUVECs were washed with sterile PBS followed by incubation for 10 min at 37°C in 1 ml TRIS (25 mM, pH 8). Plates were transferred to a freezer (-80°C) for at least 1 hour. After thawing the plates at 37°C, cells were harvested using a mechanical cell scraper and centrifuged in an Eppendorf table centrifuge at 14,000 rpm for 5 min. Cell pellets were suspended in 120 μL of assay buffer (25 mM TRIS/ PBS/0.1% BSA) and immediately assayed.

Tissue factor activity was measured using a 1-step coagulation assay (Amelung microcoagulation analyzer) by addition of 100 μL factor VIII-deficient human plasma (George-King Biomedical). Clotting time was determined at 37°C and measurement started after addition of 100 μL of 30 nM CaCl_2 _(Sigma). The reference curve for TF was obtained by reconstituting various concentrations of lipidated recombinant hTF (American Diagnostica, # 4500 L/B2) according to the manufacturer's recommendation and allowed for detection limits between 0.395 ng/mL to 25 ng/mL (7 – 470 pM/mL).

### Immunohistochemistry

HUVECs were grown to confluence on fibronectin coated two well culture slides (BIOCOAT Fibronectin, Becton Dickinson, # 354628) and exposed to IL-1 as described for the permeability assays. After fixation with 4% formaldehyde (RT, 10 min), cells were permeabilized with 1% Triton X for 10 min. A 1:200 dilution of a polyclonal VE-cadherin antibody (Santa Cruz Biotechnology, #sc-6458) was used as primary antibody and incubated at RT for 1 hour. After several washes with PBS, cells were labeled with Alexa Fluor 488 donkey anti-goat IgG (Molecular Probes, #A-11055) for 1 hour at RT. After repeated washes, the F-actin cytoskeleton was stained with Texas Red labeled phalloidin (Molecular Probes, #T-7471) for 40 min at room temperature. Slides were mounted and sealed using Gel/Mount (Biomeda, #M01). Photomicrographs were obtained with a Zeiss Axiovert 200 M inverted fluorescent microscope (Zeiss, Germany) at 400 × magnification.

## Results

### TF induction and permeability in endothelial cells by IL-1β *in vitro*

**Figure 1:**
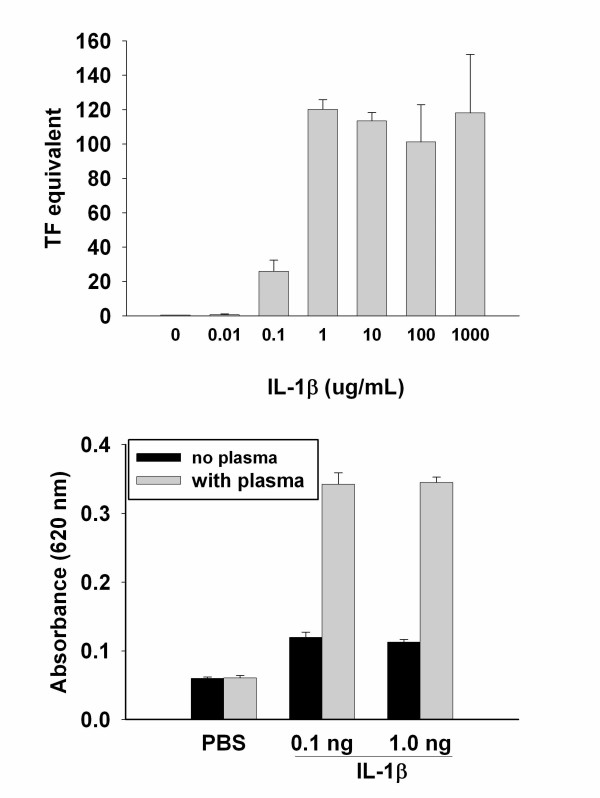
Increase in endothelial surface TF activity in response to IL-1β and dependence of IL-1β induced vascular permeability on tissue factor expression. Confluent EC monolayers were incubated for 3 hours with increasing concentrations of IL-1β and a single step coagulation assay performed to measure TF activity. A rapid increase of TF activity was observed at IL-1β of 100 pg/ml to rapidly plateau at 1 ng/ml or above (a). At concentrations of 0.1 ng and 1.0 ng/ ml, IL-1β strongly induced vascular permeability only in the presence of Factor VIII-deficient plasma and there was a modest increase in vascular permeability without plasma (b). All experiments were conducted in triplicate.

**Figure 2:**
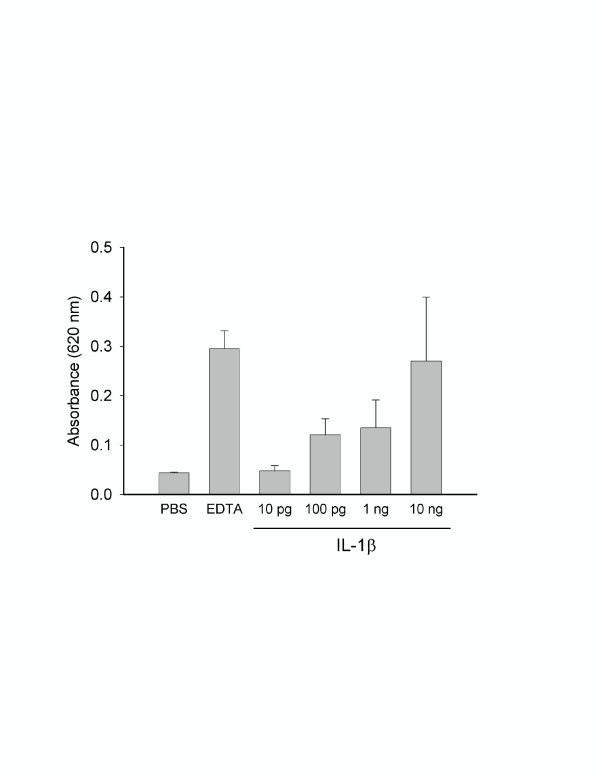
Dose dependent increase in EC monolayer permeability by 1L-1β. ECs were exposed to increasing concentrations of cytokine as described. All experiments were done in the presence of factor VIII deficient plasma.

TF induction in ECs was assessed under basal culture conditions using a single-stage coagulation assay after a 2-hour exposure to various concentrations of IL-1β. At concentrations of 100 pg/mL and higher, TF activity was markedly increased (Figure 1a). There was little TF activity measured at concentrations of IL-1β below 100 pg/mL and no further increase in TF activity was observed above 1 ng/ml. The effects of IL-1β on *in vitro *vascular permeability were assessed using a two-compartment model as described. There was only a slight increase in permeability above background (PBS control) after a 2-hour exposure to 0.1 or 1.0 ng/mL of IL-1β under basal culture conditions and exposure of ECs to factor VIII-deficient plasma alone had no effect on permeability (Figure 1b). However, at IL-1 doses that resulted in increased TF activity, IL-1 β caused a marked increase in permeability in the presence of factor VIII-deficient plasma (Figure 1b). Figure 2 shows a dose-dependent increase in EC permeability after a 2-hour incubation with IL-1β in the presence of factor VIII-deficient plasma. Permeability increased after only a 15-minute exposure to IL-1β compared to PBS control treated ECs and continued to increase with longer incubation times up to 90 minutes. However, incubation times of 2 hours or longer did not result in further increases in permeability (data not shown).

**Figure 3:**
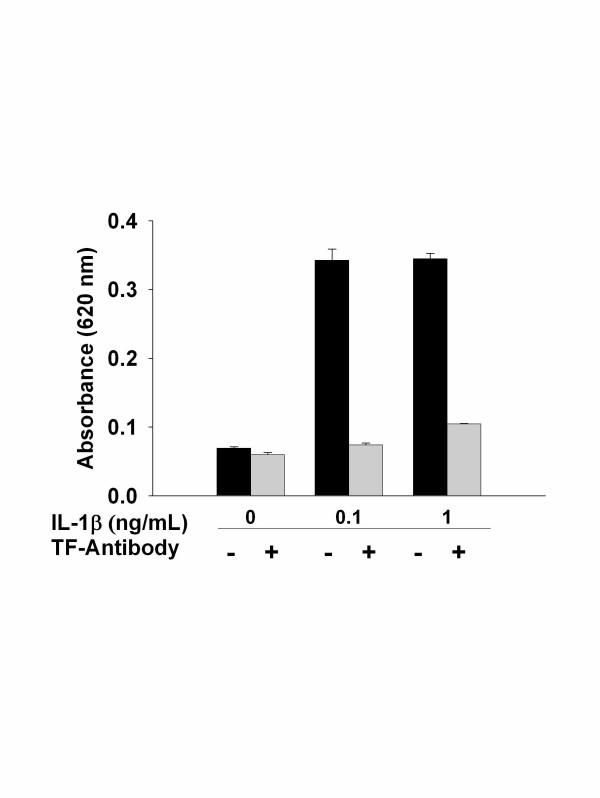
After stimulation of EC monolayers with 0.1 or 1.0 ng/ mL, IL-1β, cells were incubated with or without blocking TF-antibodies before addition of Factor VIII-deficient plasma. While permeability increased substantially in the absence of TF-blocking antibodies it could be complete abrogated by blocking TF receptors (c) demonstrating the necessity of endothelial TF activity and activation of the extrinsic coagulation cascade to induce permeability. Results were conducted in triplicate.

To test whether TF induction on ECs was responsible for the alterations in permeability by IL-1 β, experiments using pre-treatment with a neutralizing anti-TF antibody were performed. Anti-TF antibody had no independent effect on permeability. However, IL-1β induced flux of Evans Blue-bound albumin across EC monolayers was completely abrogated in the presence of TF antibody (Figure 3). Together, these data demonstrated that IL-1β induces a rapid, dose dependent increase in permeability across EC monolayers via a TF dependent mechanism.

### Activation of the extrinsic coagulation cascade by IL-1β leads to conformational changes of the EC cytoskeleton and loss of VE-cadherin complexes

**Figure 4:**
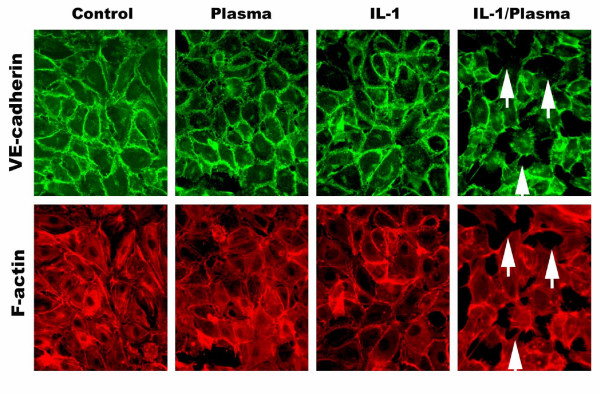
Effect on IL-1β on intercellular VE-cadherin complexes on ECs is shown. ECs were grown to confluence on fibronectin coated glass slides. Cells were stained for VE-cadherin and F-actin and visualized using an inverted fluorescence microscope. Untreated ECs form a functional monolayer without cellular gaps (control). Fluorescein labeled VE-cadherin antibodies demarcated individual cell borders whereas a Texas Red F-actin antibodies illustrate a relaxed cytoskeleton. Factor VIII-deficient plasma or IL-1 treatment alone showed similar staining patterns as untreated control cells. However, when used in combination there were large intracellular gaps seen (white arrows) in association with downregulation of VE-cadherin and the F-actin cytoskeletal filaments appear contracted and aligned in a spindle fashion.

To characterize the EC morphological changes that occur with IL-1β induced permeability we performed immunohistochemistry studies on ECs under conditions that resulted in increased permeability *in-vitro*. Figure 4 illustrates the effects of IL-1β on VE-cadherin, the major intercellular EC adhesion molecule, and F-actin cytoskeletal staining on EC monolayers. Untreated control confluent EC monolayers had homogenous VE-cadherin staining along cellular junctions with diffuse unorganized cytosolic F-actin and abundant localization adjacent to cell membranes. In the presence of factor VIII-deficient plasma alone or IL-1β alone, the EC monolayers remained intact with uniform well established intercellular tight junctions reflected by VE-cadherin and F-actin staining comparable to untreated cells. Incubation of EC monolayers with IL-1β and factor VIII-deficient plasma demonstrated markedly diminished VE-cadherin staining and associated F-actin cytoskeletal alignment particularly in areas of intercellular gap formation (Figure 4 arrows).

## Discussion

**Figure 5:**
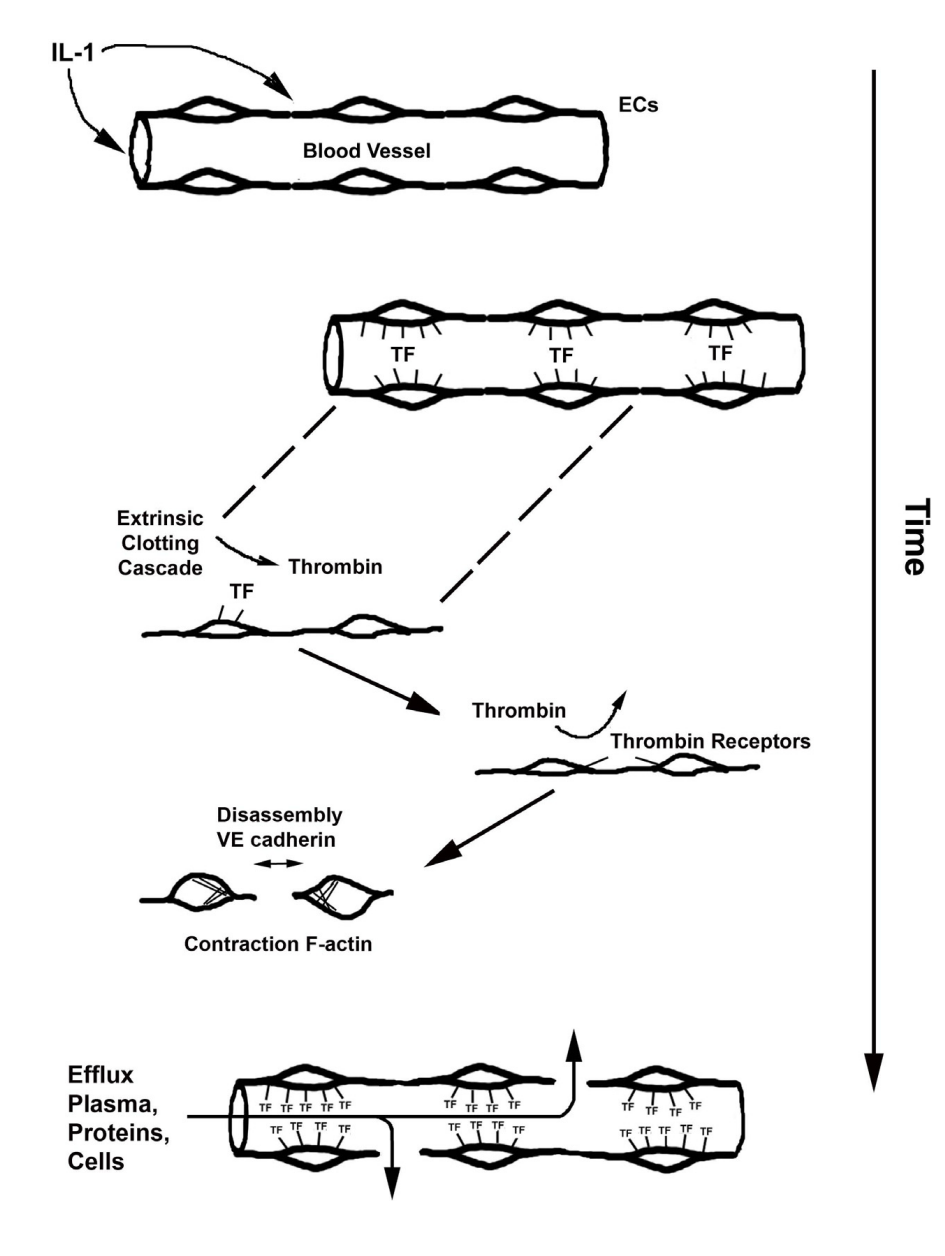
Schematic of proposed mechanism of IL-1 induced vascular permeability. Local IL-1 production results in tissue factor upregulation on EC's. In the presence of extrinsic clotting cascade factors local thrombin production activates the EC thrombin receptor and results in VE-cadherin disassembly and contraction of cellular F-actin elements. The resulting intercellular gap formation allows efflux of plasma, proteins, and inflammatory cells into the interstitium.

IL-1β exerts many of its proinflammatory effects through modulation of vascular permeability. Our data indicate that induction of tissue factor on the surface of ECs plays a central role in the regulation of vascular permeability by IL-1. Exposed endothelial surface TF, in the presence of plasma, forms complexes with coagulation factor VIIa and activates the extrinsic coagulation cascade. Local thrombin production most likely results in EC intercellular gap formation associated with decreased VE-cadherin and contraction of cytosolic F-actin fibers. We noted that in the absence of coagulation factors, IL-1β induces negligible permeability and that in the presence of factor VIII-deficient plasma, neutralizing TF antibodies completely abrogated IL-1 induced permeability. Taken together, these data indicate that IL-1 induction of TF on ECs represents a common initiating cellular event that results in both a procoagulant and permeable phenotype. Production of IL-1 at sites of acute or chronic inflammation may result in local alterations in vascular permeability mediated through the TF pathway and, to that end, modulation of TF activity may be a useful strategy to control the host inflammatory response (Figure 5).

We have previously shown that TNF shares these properties in an *in vitro *system highlighting the know widely overlapping biological activities of these two cytokines [24,25] particularly on endothelial tissue [26]. The acute loss of VE-cadherin complexes, contraction of F-actin cytoskeletal fibers, and the formation of large intercellular gaps due to TNF or IL-1 indicates that these morphological alterations are secondary to physiological EC responses rather than non-specific cell injury. EC monolayer integrity in this model is restored after the inflammatory stimuli is removed [27]. The effects are only observed in the presence of plasma, and hence, with activation of the extrinsic coagulation cascade and thrombin production.

It is interesting that the selective procoagulant and permeability effects of TNF, a cytokine with broadly overlapping biological activities with IL-1, on tumor neovasculature have been applied in the clinical treatment of cancer. When high dose TNF is administered via an isolated organ perfusion there is increased local permeability followed by selective obliteration of the neovasculature [28]. These selective effects on tumor neovasculature are thought to be important in augmenting delivery of chemotherapy, such as melphalan, to the tumor bed [29]. Our data would suggest that IL-1 might be a suitable alternative to TNF in this capacity because of the similar vascular effects and its limited toxicity compared to the other.

In summary, these experiments demonstrate that IL-1 mediates potent permeability effects on endothelial tissue that is mediated through EC surface expression of TF. To that end, TF activity may represent a novel target to ameliorate pathological inflammatory conditions mediated by this cytokine.
